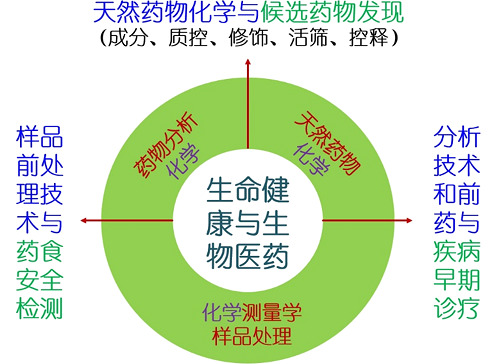# 基于超分子衍生类多孔有机聚合物的样品前处理方法研究进展

**DOI:** 10.3724/SP.J.1123.2023.09001

**Published:** 2024-06-08

**Authors:** Jingyan KANG, Yanping SHI

**Affiliations:** 中国科学院兰州化学物理研究所, 中国科学院西北特色植物资源化学重点实验室, 甘肃 兰州 730000; CAS Key Laboratory of Chemistry of Northwestern Plant Resources, Lanzhou Institute of Chemical Physics, Chinese Academy of Sciences, Lanzhou 730000, China

**Keywords:** 多孔有机聚合物, 样品前处理, 超分子衍生物, 食品分析, 环境监测, 综述, porous organic polymers, sample pretreatment, supramolecular derivatives, food analysis, environment monitoring, review

## Abstract

多孔有机聚合物是一类由有机构筑单元通过共价键连接构成的多孔材料,因具有高比表面积、可调节孔径、高度可设计性和易修饰性等特点,在样品前处理领域表现出巨大的应用潜力。设计新型功能性的构筑单元是实现目前多孔有机聚合物材料创新发展的重要因素,也是实现复杂基质中目标分子高效、高选择性分离富集的核心与关键。近年来,超分子衍生类化合物凭借其良好的主客体识别化学性质、简便易行的功能化策略以及可调节的拓扑构型,为多孔有机聚合物材料的构筑提供了新的启示和突破。本文重点归纳了不同结构类型的超分子衍生类多孔有机聚合物构筑策略,并聚焦于食品分析和环境监测,简要综述了超分子衍生类多孔有机聚合物在样品前处理领域的最新研究进展,并对该领域的发展方向进行了展望。

## Abstract (en)

Porous organic polymers (POPs) are a class of materials composed of organic building blocks usually consisting of the elements C, H, O, N, and B and other light elements connected by covalent bonds. Owing to the diversity of synthesis methods in organic chemistry, POPs can be prepared by Suzuki coupling, Sonogashira-Hagihara cross-coupling, Schiff-base condensation, Knoevenagel condensation, and Friedel-Crafts alkylation. POPs show great application potential in the field of sample pretreatment because of their large specific surface area, adjustable pore size, high tailorability, and easy modification. The design of new functional building blocks is an important factor in advancing the development of POPs and is key to the efficient separation and enrichment of target molecules in complex substrates. In recent years, supramolecular-derived compounds have provided new inspiration and breakthroughs in the construction of POPs on account of their excellent host-guest recognition properties, simple functionalization strategies, and adjustable topological configurations. The “cavitand-to-framework” approach, that is, the knitting of 0D macrocycles into hierarchical 2D or 3D POPs using suitable linkers, and extension of the research scope of supramolecular chemistry from discrete cavities to rigidly layered porous organic frameworks can lead to significant improvements in the porosity and stability of supramolecular-derived compounds. They can also provide an effective means to expand the structural diversity of POPs and generate layered structures with high porosity. This review summarizes the preparation strategies and structural characteristics of supramolecular-derived POPs with different structures, such as crown ether-based POPs, cyclodextrin-based POPs, and calixarene-based POPs. The promising applications of these materials in sample pretreatment focusing on food analysis and environmental monitoring, including epoxides, organic dyes, heavy metals, algatoxins, halogens, and antibiotic drugs, are then summarized. Next, the extraction mechanisms mainly attributed to host-guest recognition, *π-π* stacking, and hydrogen-bonding and electrostatic interactions between the supramolecular structures and analytes are described. The key role and potential advantages of the different preparation strategies and structural characteristics of these POPs in sample pretreatment are also discussed. Finally, the future prospects and remaining challenges of supramolecular-derived POPs are proposed. Supramolecular-derived POPs can not only achieve the rapid and selective extraction of target analytes during sample pretreatment but also improve the extraction effect of online solid phase extraction technologies. However, although numerous supramolecular-derived POPs have been developed, few have been applied in the field of sample pretreatment. Thus, the expansion of the application potential of more POP materials requires further exploration and research. The design and synthesis of supramolecular-derived POPs with highly selective recognition performance remains an important research direction in the field of sample pretreatment.

多孔有机聚合物(POPs)是一种具有微孔或介孔结构的多孔材料,通常由C、H、O、N、B和其他轻质元素组成^[1,2]^。基于有机化学的多样性,合成POPs材料的途径有很多种,包括Suzuki耦合、Sonogashira-Hagihara交叉耦合、Schiff-base缩合、Knoevenage缩合和Friedel-Crafts烷基化反应等^[3,4]^。通过单体的选择和特殊合成策略,可赋予POPs材料丰富的种类和功能。由于POPs具有高的比表面积、优异的热稳定性和化学稳定性、低密度性、高度可设计性和修饰性等优点,作为样品前处理材料,在食品、环境等复杂样品基质中痕量或超痕量高危污染物的富集与分离领域有显著的优势^[5⇓-7]^,主要表现在以下几个方面:第一,POPs的分子链以轻质元素为主,骨架密度低,可有效提高单位质量的目标分析物的吸附容量^[8]^;第二,优异的热稳定性和化学稳定性可拓展其在各种复杂或极端环境样品中的应用范围^[9]^;第三,高的比表面积与孔隙率可为目标分析物的吸附提供充足的吸附位点,有利于增强吸附剂与目标分析物之间的相互作用(氢键作用、范德华力等)^[10]^;第四,拓扑结构的构筑和孔径大小的可控性,有利于根据目标分析物的结构与性质调整框架和孔径,从而增强其吸附选择性^[11]^;第五,功能化修饰性有利于根据目标分析物的结构特性设计功能化材料,提高与目标分析物之间的相互作用,从而实现目标分析物的快速、高效、高选择性的富集与萃取^[12]^。以上独特的结构特性使得POPs成为样品前处理领域最具应用潜力的材料之一。然而,基于食品和环境等样品基质的复杂性,探索与发展多样并具有特异性的构筑单元,设计高效、高选择性POPs样品前处理材料仍然是实现复杂基质中目标分析物快速、精准分离与富集的重要研究方向。

在过去几十年,超分子及衍生物(包括冠醚(CE)、环糊精(CD)、杯芳烃(CA)等)作为超分子化学的重要组成部分,因具有明确的空腔结构,能够选择性地容纳客体分子或离子,被广泛应用于分子识别、分离分析、药物传递等领域^[1,13]^。然而,超分子衍生类化合物孔隙率低、化学稳定性差等缺点在一定程度上限制了其在特定环境中的应用范围。因此,利用“空腔-框架”概念^[1,14]^,即通过合适的键合作用将零维的超分子衍生类化合物“编织”成二维或三维的分层POPs,将超分子化学的研究范围从离散的空腔扩展到刚性分层的多孔有机框架中,可显著改善超分子衍生类化合物的孔隙率和稳定性,同时为扩展POPs的结构多样性和生成具有高孔隙率的分层多孔结构开辟有效途径^[14,15]^。此外,超分子衍生类化合物独特的分子或离子识别特性通过主客体化学和非共价键相互作用赋予了超分子衍生类POPs主客体识别、几何定向拓扑设计和良好的机械强度等特性,为食品和环境等复杂基质中目标分析物的样品前处理提供新的机遇^[16,17]^。

为此,本文以超分子衍生类化合物构筑单元的结构类型为切入点,重点归纳了包括冠醚、环糊精和杯芳烃等三类超分子衍生类POPs的构筑策略,并聚焦于食品分析和环境监测,系统综述了基于超分子衍生类POPs在样品前处理领域的最新研究进展。探讨不同构筑策略和结构特性在样品前处理过程中的关键作用与潜在优势,并对超分子衍生类POPs在样品前处理领域的发展方向进行了总结与展望。

## 1 超分子衍生类POPs的构筑

### 1.1 冠醚类多孔有机聚合物(CE-POPs)

冠醚作为被发现的大环宿主分子,其有富氧的结合位点及可调节的空腔结构,可与碱金属、碱土金属及有机阳离子形成稳定的络合物^[18,19]^。将冠醚作为POPs的构筑单元时,通常可以利用冠醚侧链的柔性官能团与合适的刚性结构连接单元形成拓扑设计,如Yuan等^[20]^于2021年报道一种冠醚类多孔有机聚合物的制备方法,首先将22-冠-6进行衍生化,借助其衍生物侧链的醛基官能团分别与1,4-苯二乙腈及4,4'-联苯二乙腈通过Knoevenage缩合制备得到二维层状四方结构的手性多孔有机聚合物(CCOFs)材料。值得强调的是,该CCOFs表现出优异的化学稳定性,在强酸(6 mol/L盐酸)和强碱(14 mol/L氢氧化钠)中浸泡一周仍可保持良好的结晶度。同年,An等^[21]^利用功能化的18-冠-6为构筑单元制备钴卟啉基CE-POPs材料(TAPP(Co)-B_18_C_6_-COF),冠醚构筑单元的引入有效提高了材料的亲水性和电子性能。与上述CCOFs相比,TAPP(Co)-B_18_C_6_-COF材料的制备过程更为简单,且耗时较短。此外,该材料具有较大的比表面积(906 m^2^/g),可为样品处理过程中目标分析物提供丰富的吸附位点。随后,An等^[22]^以相同的功能化18-冠-6和4,4',4″,4'″-(芘-1,3,6,8-四基)四苯胺(Py)作为有机单体,通过Schiff-base缩合反应构建了以冠醚分子为主链的新型CE-POPs材料(Py-B_18_C_6_-COF)。同时,将该合成方法拓展到功能化24-冠-8构筑单元中,制备得到具有相似拓扑结构的CE-COFs材料(Py-B_24_C_8_-COF),如图1a所示。实验结果表明Py-B_18_C_6_-COF与Py-B_24_C_8_-COF均具有良好的结晶度、高的比表面积与优异的化学稳定性,且分别对碱金属K^+^和Cs^+^展现出良好的吸附性能,这可能归因于冠醚结构单元18-冠-6与24-冠-8的引入及其与目标碱金属之间的尺寸匹配效应。

**图 1:**
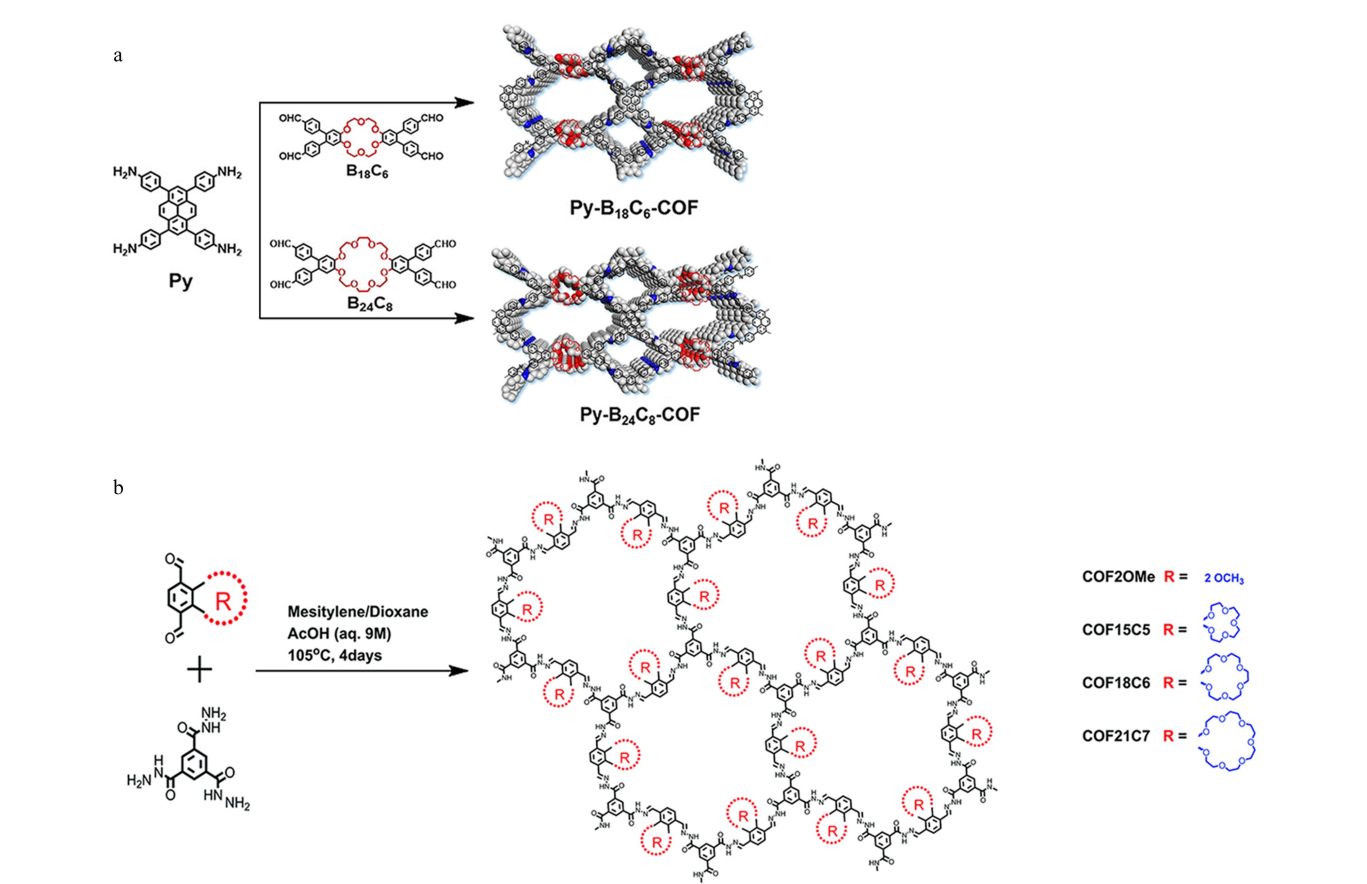
CE-POPs的合成示意图

上述CE-POPs的合成是以功能化冠醚构筑单元为主链引入拓扑结构中的。除此之外,冠醚构筑单元还可以以侧链的方式引入POPs的构筑中。如Yuan等^[23]^利用3,6-二醛基-邻苯二酚为原料制备得到具有环冠醚侧链结构的醛基配体,然后将其与1,3,5-三羧基肼通过亚胺缩合反应制备得到一系列半刚性冠醚侧链的共价有机框架材料(COFs),见图1b。将环冠醚侧链修饰在COFs空腔内壁,半刚性冠醚侧链会随着晶体结构堆叠而紧密重叠,有效限制了主链围绕腙键分子的内旋转,降低了腙键连接单元的构象自由度。然而,由于冠醚侧链在COFs空腔内部占据一定的体积,导致这一系列半刚性冠醚侧链COFs的比表面积偏低。

目前,CE-POPs材料的相关报道较少,其合成主要是利用功能化冠醚表面的醛基与氨基或氰基配体通过缩合反应合成。制备得到的CE-POPs具有良好的化学稳定性,同时兼具冠醚分子的主客体识别性能。然而,目前合成高结晶性CE-POPs仍然具有一定的挑战,这主要是因为在结晶过程中冠醚构象的灵活性所致^[22]^。

### 1.2 环糊精类多孔有机聚合物(CD-POPs)

环糊精是一类锥形环状低聚糖,通常由天然材料(如玉米、小麦、马铃薯等)经酶降解获得。环糊精主要有3种天然结构类型,即*α*-环糊精、*β*-环糊精、*γ*-环糊精,它们分别含有6个、7个、8个重复的葡萄糖醛酸单元,由*α*-1,4糖苷键连接而成。环糊精的亲水性外表使其易溶于水溶液中,而空腔内由于受到C-H键的屏蔽作用形成疏水区,可容纳金属离子、金属氧化物或有机小分子,在手性分离、重金属或有机污染物的吸附、分离及药物递送方面得到广泛应用^[24,25]^。特别是位于环糊精2,3,6位的羟基官能团易被选择性地功能化,可作为螯合或亲核位点,使其更易于引入到POPs的拓扑结构中。传统的环糊精聚合物是将环糊精通过环氧氯丙烷或者其他的小分子交联剂进行交联,利用这种方式制备的聚合物除了环糊精固有的空腔外,几乎没有拓扑结构孔隙,其比表面积较低(<100 m^2^/g),热稳定性较差,严重限制了材料的吸附性能和萃取效率(见图2a)^[26]^。因此,一些新型的交联剂和合成策略相继被探索与开发,以提高CD-POPs的孔隙率和比表面积。目前,基于Friedel-Crafts烷基化反应和亲核芳香取代反应的合成方法可以有效地解决这一难题。

**图 2:**
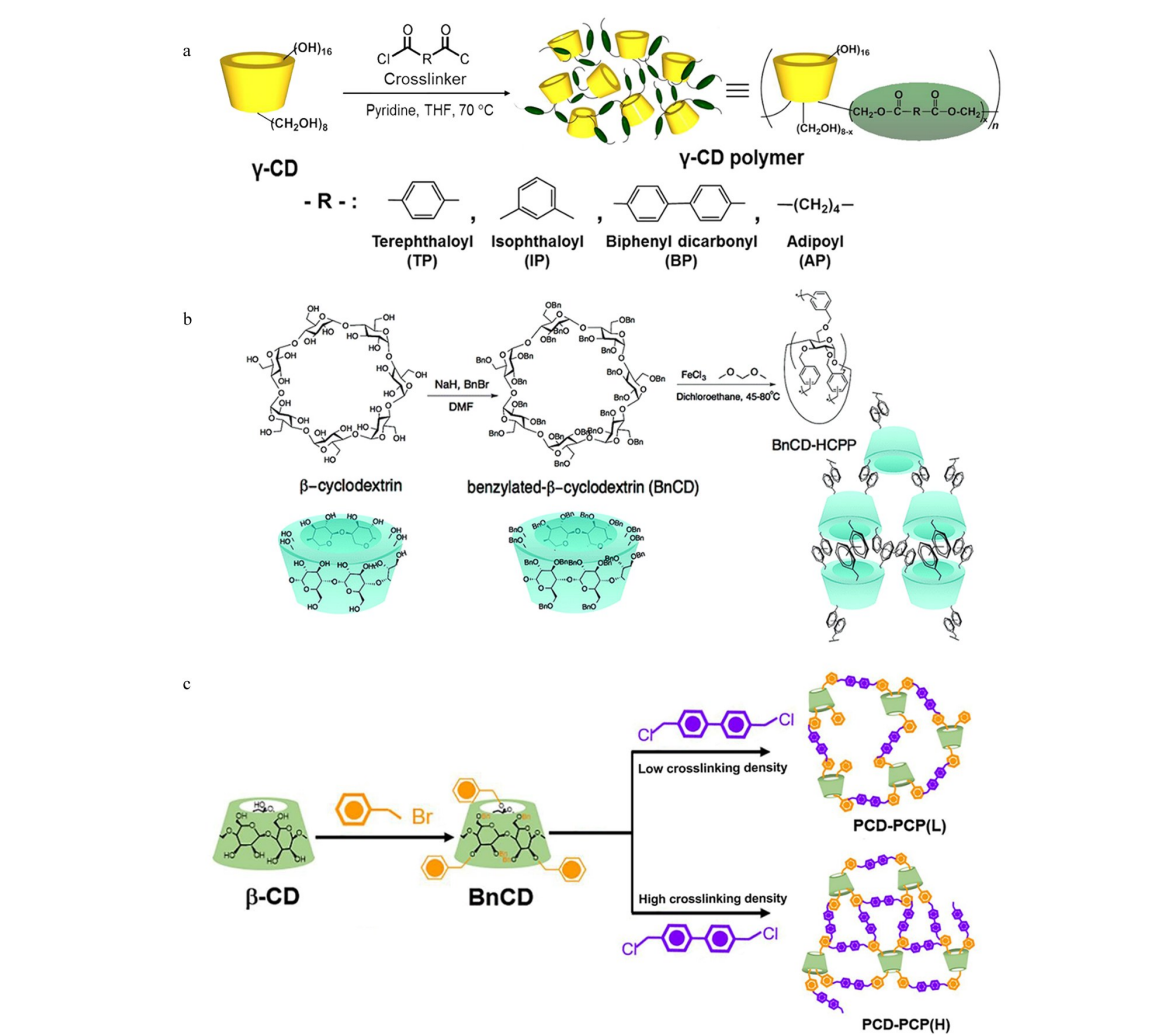
CD-POPs的制备示意图

基于Friedel-Crafts烷基化反应制备CD-POPs的主要方式是合成完全苄基化的*β*-环糊精(BnCD),以甲醛二甲基缩醛(FDA)为交联剂、氯化铁为Lewis酸催化剂进行超交联,得到CD-POPs(BnCD-HCPP),如图2b所示^[27]^。与环氧氯丙烷反应制备得到的环糊精聚合物相比,BnCD-HCPP具有优异的比表面积(1225 m^2^/g),这主要归因于刚性和扭曲苯环之间的填充作用及*β*-环糊精固有的空腔。此外,基于BnCD-HCPP良好的稳定性,将其成功应用于芳香族污染物的吸附,并展现出良好的吸附性能。Huang等^[28]^以4,4'-双(氯甲基)作为交联剂,利用相似的Friedel-Crafts烷基化反应制备得到富苯基CD-POPs。在合成过程中通过交联剂的浓度对材料的交联程度进行控制和调节,得到具有高低两种密度交联的多孔聚合物PCD-PCP(L)和PCD-PCP(H)(见图2c)。实验表明高密度交联的CD-POPs具有高的比表面积(1098 m^2^/g)和良好的化学稳定性,可成功应用于污水中2,4,6-三氯苯和双酚A的吸附。

除了上述介绍的Friedel-Crafts烷基化反应,以刚性氟化芳香类化合物为交联剂的芳香族亲核取代反应(SNAr)也是制备CD-POPs的重要方法。Alsbaiee等^[29]^在利用亲核芳香取代反应制备具有高比表面积的CD-POPs方面取得重大进展(见图3a)。该方法利用*β*-环糊精表面的羟基与刚性芳香族四氟对苯二甲酸(TFN)进行SNAr反应制备得到CD-POPs(TFN-CDP)。重要的是,基于*β*-环糊精的主客体识别效应和TFN-CDP的介孔累积效应,该材料在各种有机污染物的吸附方面表现出优异的性能,吸附速率约是活性炭和无孔*β*-环糊精聚合物的15~200倍,且其具有良好的重复使用性。除*β*-环糊精外,*α*-环糊精也具有相似的功能与性质,且其具有更好的亲和力。例如,Dolai等^[30]^发展了一种基于*α*-环糊精的多孔共价分子印迹聚合物(MIP),其制备方式是在*α*-环糊精和邻苯二甲酸二丁酯(DBP)的主客体络合过程中以TFN为交联剂通过SNAr反应对*α*-环糊精进行交联,DBP在交联过程中控制*α*-环糊精的相互取向。聚合后将DBP去除即得到对DBP具有特异选择性的分子印迹聚合物,其制备过程如图3b所示。当*α*-环糊精、TFN与DBP之间的比例达到最佳物质的量之比(1∶3∶9)时,该分子印迹聚合物的印迹因子达2.6,最佳吸附容量为22 mg/g。

**图 3:**
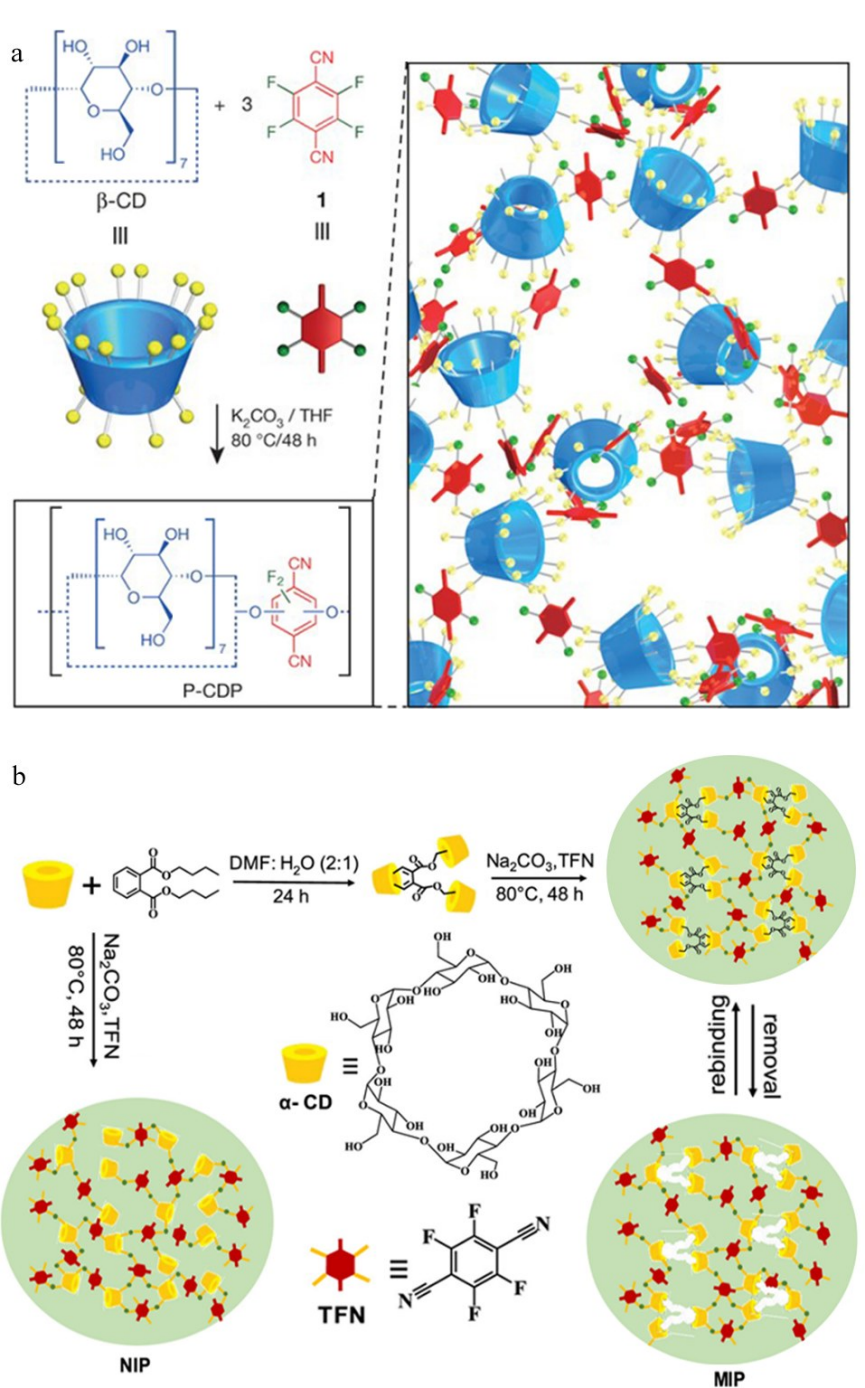
基于亲核芳香取代反应制备CD-POPs的示意图

用环糊精作为POPs构筑单元的合成策略还有很多,如酯交联^[31]^、酰亚胺交联^[32]^、异丁酸脂交联^[33]^等方式,在此我们不再一一赘述。总之,将环糊精引入POPs中,可使CD-POPs兼具环糊精主客体识别与POPs拓扑结构的协同功效;通过选取不同的构筑单元与合成策略,可实现不同结构与性能CD-POPs的设计与调控,相关的制备方法已发展得较为成熟。

### 1.3 杯芳烃类多孔有机聚合物(CA-POPs)

杯芳烃是一类由亚甲基桥连苯酚单元所构成的大环结构化合物,因其具有大小可调节的疏水空腔,且疏水空腔被易改变的极性和非极性环包围,可作为超分子化学中不同类型客体分子的强大受体^[34,35]^。与冠醚、环糊精相比,杯芳烃是一类更具有广泛适用性的大环结构分子,被称为继冠醚和环糊精之后的第三代主体化合物。尤其是杯[4]芳烃衍生物,可以形成锥形、部分锥形、1,2-交替和1,3-交替构象^[11]^,且其结构中的极性苯酚单元、非极性芳香环及亚甲基桥连键等易被修饰为多功能的超分子衍生物。此外,杯芳烃的刚性结构有利于通过共价键的形式将其引入POPs中,为CA-POPs的设计与制备提供结构基础。

传统的杯芳烃是作为侧链结构被引入到聚合物中的。2017年,Shetty等^[36]^报道了以杯芳烃为主链的POPs的合成方法,以四溴杯[4]芳烃与1,4-二乙基苯为构筑单元,通过Sonogashira-Hagihara交叉耦合反应制备得到CA-POPs (CaIP),如图4a所示。利用该方式制备得到的CA-POPs中引入富*π*电子的炔基官能团,可增强聚合物骨架结构的刚性和共轭性,同时赋予材料高比表面积(596 m^2^/g)和较大的孔体积(0.73 cm^3^/g)。随后,Shetty等^[37]^利用3种不同的乙炔功能化连接单元,通过Sonogashira-Hagihara交叉耦合进一步扩展了CA-POPs的结构类型(见图4b)。通过红外光谱和核磁共振证实CA-POPs的成功制备,由于在结构中引入更多的乙炔基团,该类CA-POPs具有更大的比表面积(596~759 m^2^/g)。近期,我们也利用Sonogashira-Hagihara交叉耦合设计制备了一种新型的偶氮苯基杯[4]芳烃多孔有机聚合物材料,并将其应用于食品中三苯甲烷类染料的快速富集和分离分析^[14]^。尽管三维CA-POPs的制备方法已有报道,然而由于杯芳烃非平面结构且构象灵活,将其作为构筑单元引入到二维POPs中仍具有一定的挑战性。为此,Shetty等^[38]^报道了一种无模板法,利用5,11,17,23-四溴杯芳烃与4,4-二乙基-1,1'-联苯为构筑单元,通过溶剂热法制备得到二维柔性CA-POPs(CX4-NS),如图4c所示。在CX4-NS中,杯芳烃采用1,2-交替构象构筑拓扑结构,形成菱形结构单元,宏观上呈现为平行层紧密堆积的3.52 nm的纳米片。此外,杯芳烃中的羟基基团也可被功能化修饰后引入POPs中,如Shetty等^[39]^利用含硫醚冠的杯[4]芳烯衍生物与四乙炔芘为构筑单元合成富硫醚冠的杯芳烃基多孔聚合物S-CX4P(见图4d)。

**图 4:**
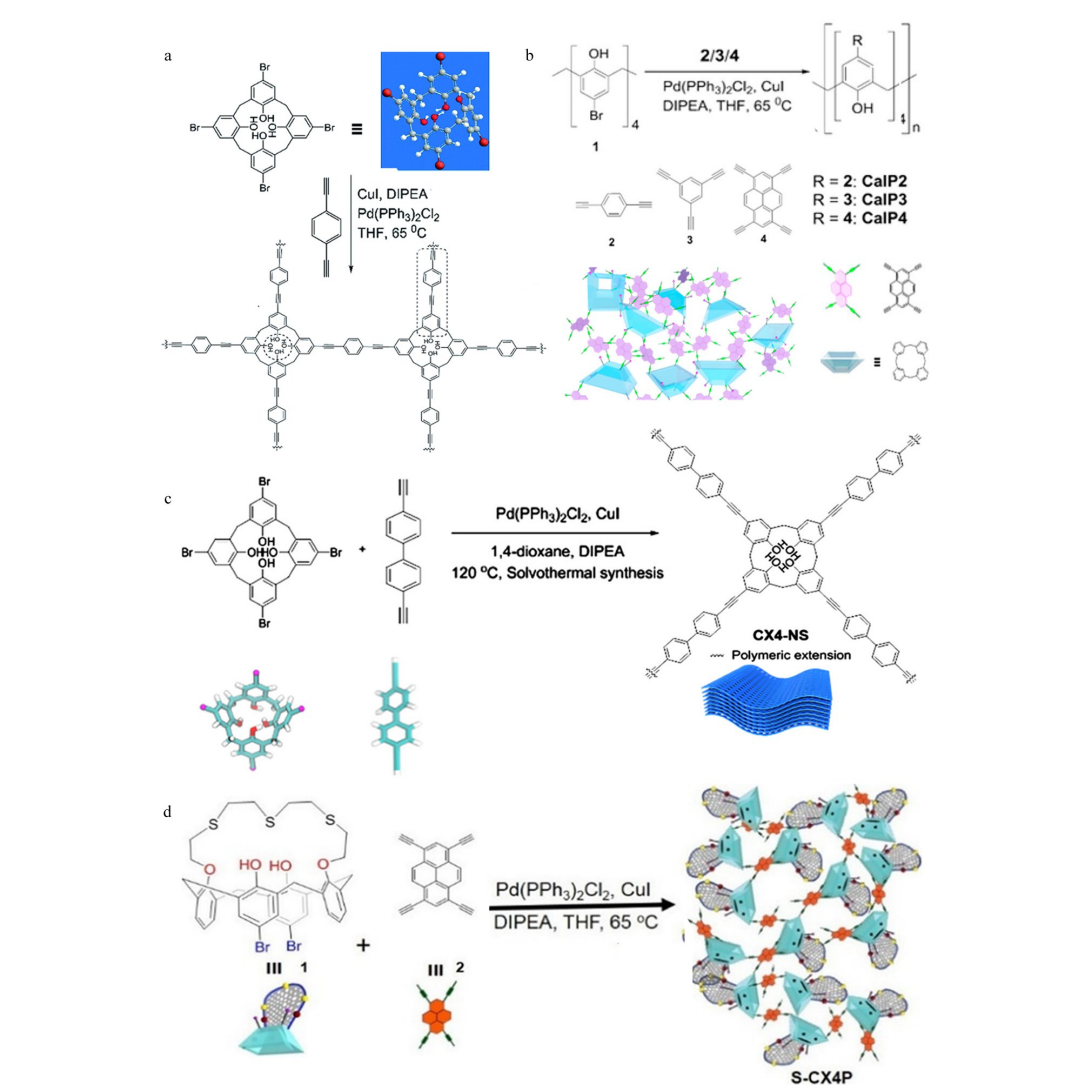
基于Sonogashira-Hagihara反应制备杯[4]芳烃类多孔有机聚合物

除Sonogashira-Hagihara交叉耦合反应外,重氮偶联反应和亲核芳香取代反应也是合成CA-POPs的重要途径。Skorjanc等^[40]^报道了以杯[4]芳烃的硝基衍生物(*p*TNC4A)与两种不同的紫罗兰二胺为原料,通过重氮耦合反应合成两种具有氧化还原性的CA-POPs(COP1^++^和COP2^++^,见图5a)。虽然COP1^++^和COP2^++^属于非晶型结构,其比表面积较小(分别为17.9 m^2^/g和51.8 m^2^/g),但将其用于水中偶氮染料的吸附时吸附效率可接近100%。Zhang等^[41]^报道了4种CA-POPs(CalCOP),其主要是通过具有不同烷基链的氨基杯[4]芳烃和三嗪衍生物之间的亲核芳香取代反应制备得到。BET表征发现CalCOP材料的比表面积随着烷基链长度从乙基到丁基的增加而降低,这可能是由于聚合过程中杯芳烃的构象发生了变化。Zhang等^[42]^报道了一种基于1-溴甲基-4-羟基杯[4]芳烃和三(4-咪唑基苯基)胺的新型阳离子CA-POPs,该聚合物也是通过类似的亲核芳香取代反应合成的(见图5b)。

**图 5:**
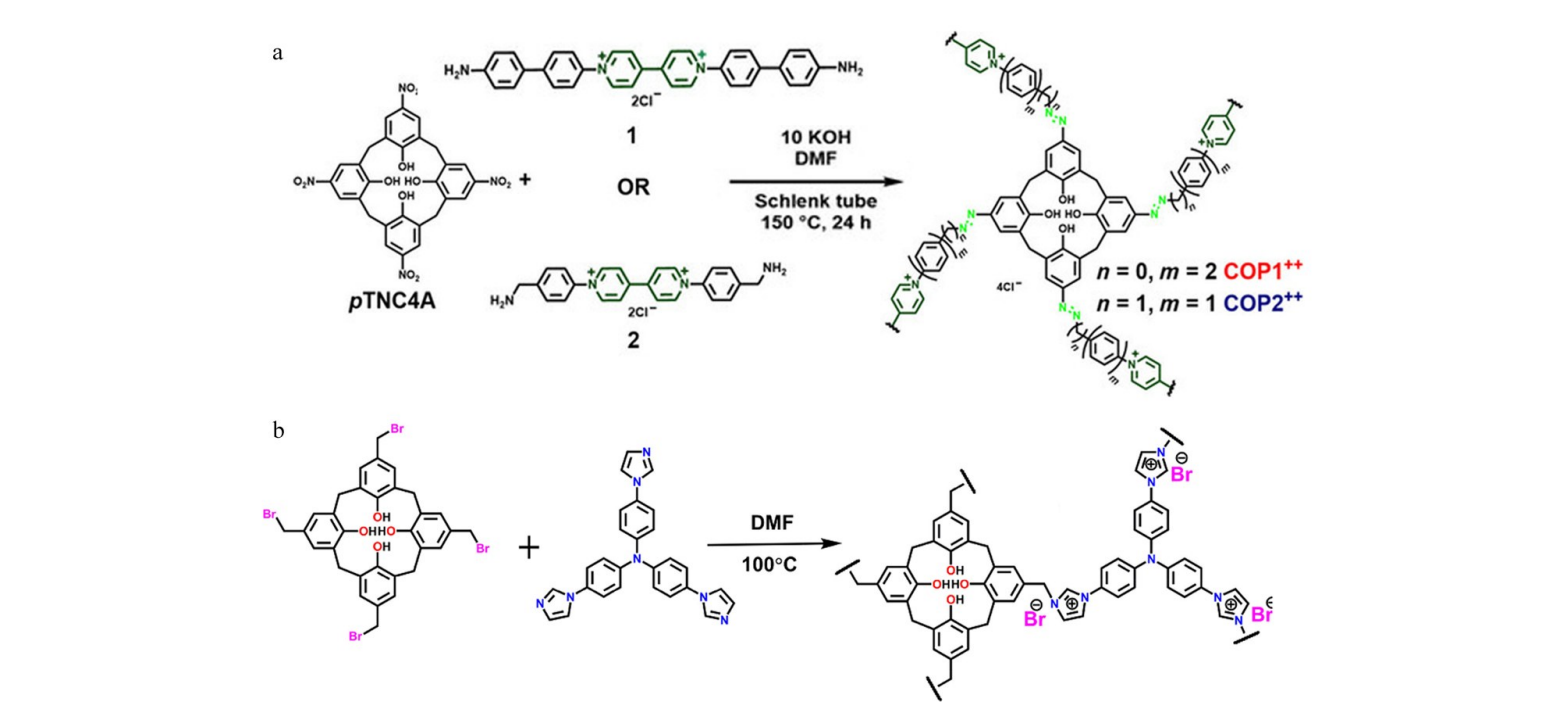
基于重氮偶联反应制备杯[4]芳烃类多孔有机聚合物

到目前为止,以杯芳烃及其衍生物为构筑单元的共价有机聚合物主要通过Sonogashira-Hagihara交叉耦合、重氮偶联反应和亲核芳香取代反应3种方法制备得到,其中基于交叉耦合制备杯芳烃类多孔有机聚合物是最常用的方法,且得到的聚合物比表面积较大,结构类型及有效官能团更丰富,可为样品前处理材料提供更有利、更广泛的设计思路。

## 2 超分子衍生类POPs在样品前处理中的应用

超分子衍生类化合物的引入使得POPs的选择性吸附与分离的定向设计与制备成为可能。由于超分子化合物独特的主客体识别性能和空腔结构的可调节性,故可根据不同类型目标分析物的结构特性和化学性质设计具有高效、高选择性分离与富集性能的超分子衍生类POPs。随着超分子衍生类POPs的设计与发展,不同构筑单元合成的超分子衍生类POPs在样品前处理中的应用将会越来越多,由于所制备得到的POPs通常具有较大的比表面积与特定的官能团等优势,该类材料在食品、环境等领域中痕量或者超痕量无机或有机污染物的样品前处理与分析检测方面将有广阔的应用前景。

### 2.1 食品分析

食品安全问题是关系着人民生命健康与社会稳定的重要因素,一直备受关注。近年来,全球由于食品不合格引发的食品安全事件日趋频繁,如何能够快速、灵敏地实现各类食品中有害成分的分析检测已成为分析领域亟待解决的问题^[43,44]^。然而,食品分析在种类繁多、含量低且基质复杂的有害成分检测时面临着巨大的挑战。基于超分子衍生类POPs的样品前处理技术在提升食品中痕量或超痕量目标分析物的纯化、分离与富集效果方面有显著的作用^[45,46]^。如Xie等^[47]^提出一种核-壳结构的磁性磺氨基杯[6]芳烃POPs作为磁性固相萃取剂,结合超高效液相色谱-串联质谱检测技术(UHPLC-MS/MS)用于罐头食品中环氧衍生物的固相萃取与分析检测(见图6)。由于吸附剂具有良好的主客体识别性能、丰富的结合位点及合适的空腔大小,对目标分析物环氧衍生物展现出优异的萃取性能;将其用于罐头类饮料、鱼、肉和牛奶等不同食品样品中13种环氧衍生物的分析检测,其检出限为0.0072~0.023 ng/g,回收率为74.9%~118%。Li等^[48]^制备了一种*β*-环糊精类POPs材料(P-CDP),以其作为萃取剂,并结合高效液相色谱-紫外检测(HPLC-UV)技术用于橙汁中双酚A、双酚F及双酚AF的固相萃取与分析检测。实验结果表明,双酚类目标分析物的检出限为0.3 ng/mL。Li等^[49]^采用简单的方法制备*γ*-环糊精类POPs,将其作为一种绿色、可再生固相萃取剂用于磺胺噻唑、磺胺甲氧基胺、磺胺嘧啶、磺胺二甲氧嘧啶及磺胺喹啉等5种磺胺类化合物的选择性萃取,并结合HPLC实现了肉类样品中磺胺类化合物的快速、灵敏分析。

**图 6:**
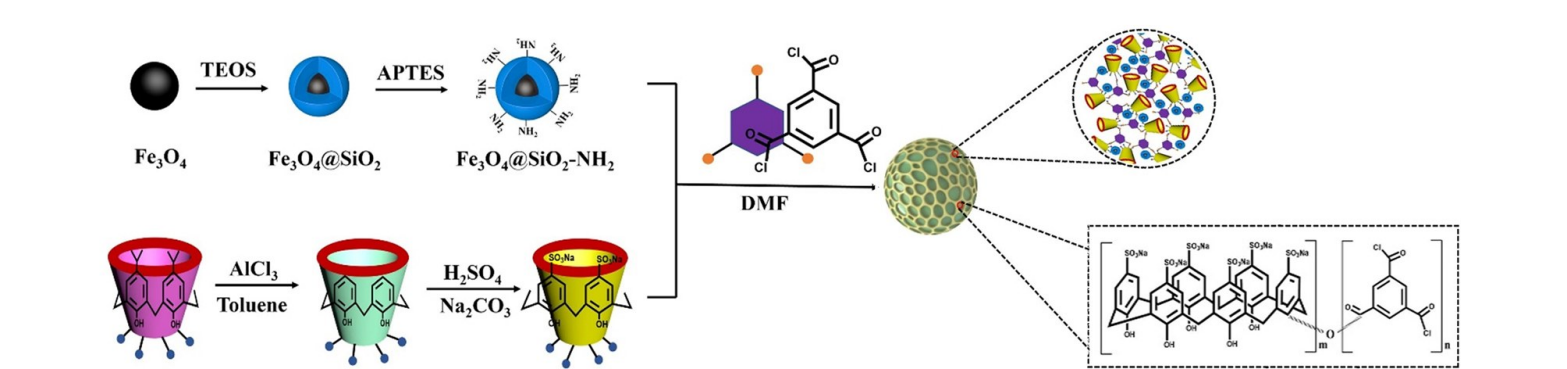
核-壳结构的磁性磺氨基杯[6]芳烃共价交联有机聚合物制备示意图^[47]^

以超分子衍生类POPs为样品前处理材料的报道还有很多,因制备的超分子衍生类POPs具有较大的比表面积和丰富的吸附位点,主要是通过超分子结构中的主客体识别作用、*π-π*堆积、氢键作用、静电作用等的协同作用实现对目标分析物的高效分离与富集。随着不同结构、不同类型超分子衍生类POPs材料的设计与开发及其在结构与性能方面的优势,在食品中不同类型目标分析物的样品前处理领域展现出极大的应用潜力,可为推动食品领域精准分离分析技术的发展奠定基础。

### 2.2 环境污染物分析

现阶段,我国高度重视环境污染治理工作,并采用相关措施不断完善污染监测标准,加强对各类污染物的检测力度。造成环境污染的污染物种类有很多,如重金属、耗氧物质污染及植物营养物质污染等^[50]^。而环境污染物的分析与检测就成为监测环境受污染程度的直接证据,具有十分重要的意义^[51]^。基于超分子衍生类POPs的样品前处理材料因具有良好的化学稳定性、较大的比表面积及丰富的萃取位点等特性,近几年在环境污染物分析领域已崭露头角^[52]^。Zhou等^[53]^以八氟萘和十氟联苯作为交联剂,通过简单而温和的方法制备了两种新型CA-POPs(POP-8F和POP-10F);将其作为萃取剂实现了水体中罗丹明B、结晶紫、亚甲基蓝等阳离子染料的高效吸附,吸附含量高达2433 mg/g。Duan等^[54]^合成了功能化磁性*β*-CD-POPs,将其用于环境水中苏丹染料的高选择性萃取,并结合HPLC建立了水环境苏丹染料的分析检测方法,检出限为0.013~0.054 ng/mL,相应的回收率为85.8%~102.8%。

除有机小分子染料外,超分子衍生类POPs还可用于藻毒素类污染物的萃取与分析。例如,Zhang等^[55]^以合成的磁性CD-POPs作为磁性固相萃取剂,结合HPLC-MS/MS建立了一种用于水环境中微囊藻毒素的简便、高效、灵敏的分析检测方法。建立的分析方法具有较宽的线性范围(2.0~1000 pg/mL)、较低的检出限(1.0~5.0 pg/mL)和良好的重复性(相对标准偏差(RSD)<9.4%)。为进一步探究POPs在环境中微囊藻毒素的应用范围,该团队利用*γ*-环糊精为构筑单元合成了一种磁性环糊精类多孔聚合物(Fe_3_O_4_@PDA@*γ*-CDP),如图7所示^[56]^。制备得到的固相萃取剂具有良好的分散性和较高的亲和力,对水溶液中微囊藻毒素具有优异的萃取性能,结合HPLC-MS/MS检测技术建立了淡水和海水中微囊藻毒素的快速、灵敏检测方法。此外,通过调整超分子衍生物的结构、连接单元的类型及合成方式制备得到的超分子衍生类POPs作为样品前处理材料还被用于环境样品中重金属离子^[39]^、卤素^[57]^和抗生素类药物^[58]^等的高效、特异性萃取与分离。超分子衍生类POPs在样品前处理领域的应用见表1,表明超分子衍生类POPs在样品前处理领域具有良好的应用前景。

**图 7:**
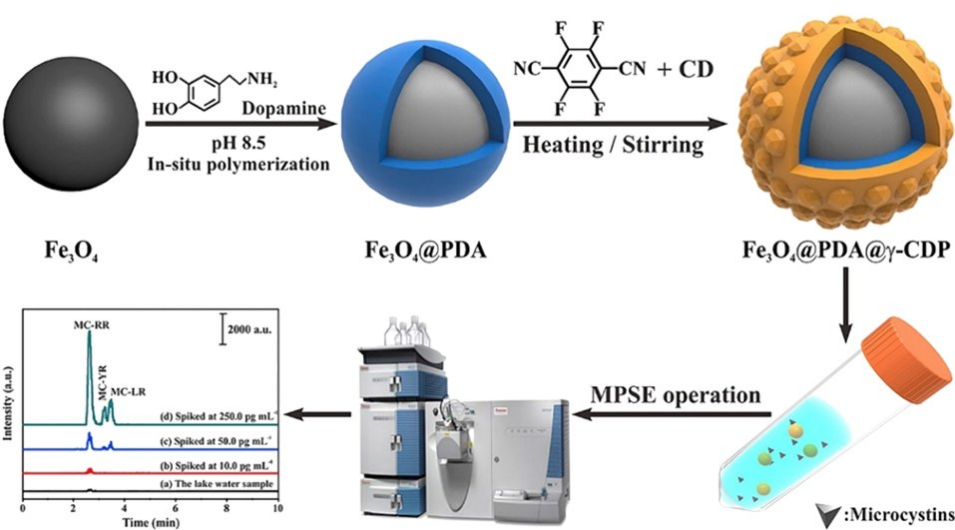
磁性环糊精类多孔有机聚合物Fe_3_O_4_@PDA@ *γ*-CDP的制备及其固相萃取应用示意图^[56]^

**表 1:** 超分子衍生类POPs在样品前处理领域的应用

Extraction material	Analytes	Detection method	Linear range/(ng/mL)		LODs/(ng/mL)		Ref.
Fe_3_O_4_@pTMP-SC6A	epoxy derivatives	UPLC-MS/MS	0.05	-100	0.0072	-0.023	[47]
P-CDP	bisphenols A, F, AF	HPLC-UV	0.0001	-0.12	0.3		[48]
CD-MOF	sulfonamides	HPLC	10	-1000	0.32	-1.7	[49]
MPCDPs	Sudan dyes	HPLC-DAD	0.1	-20	0.013	-0.054	[54]
Fe_3_O_4_@SiO_2_@P-CDP	microcystins	HPLC-MS/MS	0.002	-1	0.001	-0.005	[55]
Fe_3_O_4_@PDA@γ-CDP	microcystins	HPLC-MS/MS	0.001	-1.0	0.0008	-0.002	[56]
CDP	quinolones	HPLC	25	-5000	2.67	-5.5	[58]

Fe_3_O_4_@pTMP-SC6A: Fe_3_O_4_ modified sulfonatocalix[6]arene covalent cross-linked porous polymer; P-CDP: porous *β*-cyclodextrin polymer; CD-MOF: cyclodextrin metal-organic framework; MPCDPs: magnetically modified porous *β*-cyclodextrin polymer; Fe_3_O_4_@SiO_2_@P-CDP: magnetic porous *β*-cyclodextrin polymer; Fe_3_O_4_@PDA@*γ*-CDP: magnetic *γ*-cyclodextrin polymer; CDP: porous *β*-cyclodextrin polymer.

## 3 总结与展望

本文简要介绍了基于冠醚、环糊精及杯芳烃三类超分子衍生类POPs的主要合成策略,包括Suzuki耦合、Sonogashira-Hagihara交叉耦合、重氮耦合、Schiff-base缩合、Knoevenage缩合和Friedel-Crafts烷基化反应等,并阐述了利用不同构筑单元和合成策略制备的POPs材料的结构和性能特征。将超分子衍生类化合物引入POPs的拓扑结构中,可显著改善超分子衍生类化合物的孔隙率和稳定性,同时借助超分子衍生类化合物独特的分子或离子识别特性赋予了POPs主客体识别、几何定向拓扑设计和良好的机械强度等特性,为样品前处理新材料的设计与开发奠定了理论基础。此外,基于超分子衍生类POPs较高的比表面积、良好的化学稳定性、可调节的拓扑结构及易功能化修饰等优势,该类材料在食品和环境的样品前处理领域展现出极大的应用潜力。将超分子衍生类POPs作为固相萃取载体,不仅可以实现样品前处理过程中目标分析物的快速和选择性萃取,而且可提高在线固相萃取技术的萃取效果。然而,相对于目前已开发的众多超分子衍生类POPs而言,应用于样品前处理领域的超分子衍生类POPs依然较少,更多POPs材料的应用潜力有待研究者的进一步探索与挖掘。目前,超分子衍生类POPs在样品前处理领域的研究还处于初始阶段,结合不同类型待测分析物的结构和化学特性,设计或筛选具有高选择性识别性能的超分子衍生类POPs仍是该材料在样品前处理领域的重要研究方向。

## 作者团队简介

药物化学成分与分析技术研究组隶属于中国科学院兰州化学物理研究所。自2001年以来,研究组面向人民生命健康,重点针对生物医药,主要从事化学测量学领域的天然药物化学成分的鉴定与分析、复杂体系的样品前处理材料设计制备与色谱分析、药物分子递送与成像分析等创新性研究工作,构建食品安全、药品安全、疾病诊疗等技术创新体系。研究组具备完善的药物分离分析平台,与国内外科研院所建立了长期良好的合作关系。

**Figure:**
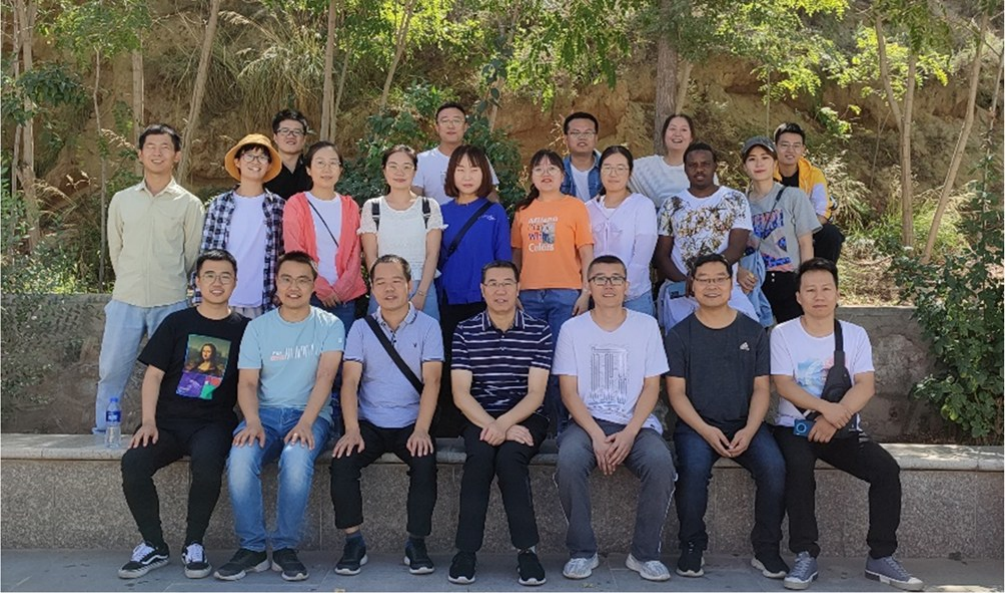


### 科研团队与精神

**Figure:**
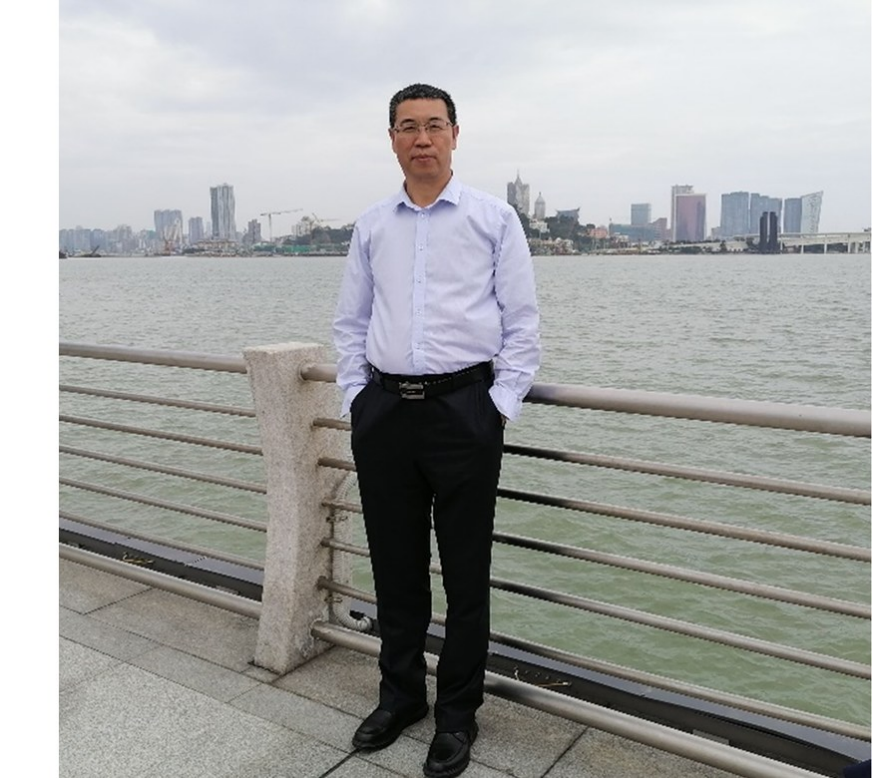


**研究组组长:**师彦平研究员,国务院政府特殊津贴专家,中国科学院引进国外杰出人才,中国科学院特聘研究员,甘肃省领军人才,甘肃省特聘科技专家。个人网页 http://people.ucas.edu.cn/~cnprshiyp。

**研究组副组长:**哈伟研究员,中国科学院特聘研究员,中国科学院西部之光人才,中国科学院青年创新促进会会员。

**研究组职工和研究生:**研究员2名,副研究员3名,助理研究员等5名,博士和硕士研究生10余名。

**团队精神:**勤学 苦练 勇于创新, 求真 务实 追求卓越, 友爱 协作 永续发展, 保国 为家 坚定信念。

### 科研项目及成果

研究组先后承担国家和省部级科研项目40余项。在*TrAC-Trends in Analytical Chemistry*, *Analytical Chemistry*, *Analytica Chimica Acta*, *Talanta*, *Journal of Chromatography A*, *Natural Products Reports*, *Organic Letters*, *Food Chemistry*, *Green Chemistry*, *Chemical Communications*, *ACS Applied Materials & Interfaces*等期刊上发表科研论文400余篇,其中高水平SCI收录论文300余篇。申请中国发明专利78件,其中授权专利38件。撰写《单萜和倍半萜化学》专著1部,参编《核法证学技术在海关打击放射性物质与特殊核材料跨境走私中的应用》著作1部。培养研究生80余名, 其中10余名博士生获得中科院冠名奖学金和国家博士研究生奖学金。

### 学术奖励与荣誉

获国家教育部科技进步二等奖2项,甘肃省自然科学二等奖4项、三等奖1项。中国科学院优秀教师和中国科学院朱李月华优秀教师。

### 研究领域与方向

面向人民生命健康与生物医药领域。研究方向:1. 天然药物资源的化学成分; 2. 样品前处理材料与分析技术; 3.药物分子递送与成像分析。

**Figure:**